# Real-Time Implementation of EEG Oscillatory Phase-Informed Visual Stimulation Using a Least Mean Square-Based AR Model

**DOI:** 10.3390/jpm11010038

**Published:** 2021-01-11

**Authors:** Aqsa Shakeel, Takayuki Onojima, Toshihisa Tanaka, Keiichi Kitajo

**Affiliations:** 1 CBS-TOYOTA Collaboration Center, RIKEN Center for Brain Science, Wako 351-0198, Japan; aqsashakeel@gmail.com (A.S.); takayuki.onojima@riken.jp (T.O.); tanakat@cc.tuat.ac.jp (T.T.); 2 Department of Electronic and Information Engineering, Tokyo University of Agriculture and Technology, Tokyo 184-8588, Japan; 3 Division of Neural Dynamics, Department of System Neuroscience, National Institute for Physiological Sciences, National Institutes of Natural Sciences, Okazaki 444-8585, Japan; 4 Department of Physiological Sciences, School of Life Science, The Graduate University for Advanced Studies (SOKENDAI), Okazaki 444-8585, Japan

**Keywords:** electroencephalography (EEG), brain state-dependent stimulation, closed-loop, autoregressive (AR) model, Yule–Walker (YW) method, least mean square (LMS) method, alpha oscillation, Instantaneous phase

## Abstract

It is a technically challenging problem to assess the instantaneous brain state using electroencephalography (EEG) in a real-time closed-loop setup because the prediction of future signals is required to define the current state, such as the instantaneous phase and amplitude. To accomplish this in real-time, a conventional Yule–Walker (YW)-based autoregressive (AR) model has been used. However, the brain state-dependent real-time implementation of a closed-loop system employing an adaptive method has not yet been explored. Our primary purpose was to investigate whether time-series forward prediction using an adaptive least mean square (LMS)-based AR model would be implementable in a real-time closed-loop system or not. EEG state-dependent triggers synchronized with the EEG peaks and troughs of alpha oscillations in both an open-eyes resting state and a visual task. For the resting and visual conditions, statistical results showed that the proposed method succeeded in giving triggers at a specific phase of EEG oscillations for all participants. These individual results showed that the LMS-based AR model was successfully implemented in a real-time closed-loop system targeting specific phases of alpha oscillations and can be used as an adaptive alternative to the conventional and machine-learning approaches with a low computational load.

## 1. Introduction

Closed-loop neuroscience is gaining more attention with ongoing technological and innovative advances that enable complex feedback loops to be executed with millisecond resolution on hardware. With regard to brain mechanics, much has been learned about stimulation in an open-loop manner using a pre-defined stimulus, such as the determination of input–output characteristics and how these are possibly modified. This open-loop approach has been quite productive in the field of non-invasive brain stimulation (NIBS), facilitating major developments in pharmacological understanding, as well as in the understanding of the functional basis of cortical dynamics [1,2].

An experiment may be viewed as a “closed-loop” when output from the brain becomes future input to the brain. By creating a causal relationship between the stimulus generator and the measured output, one can possibly “close the loop” [3] in laboratory settings. In reality, this can be achieved when a presented stimulus depends on the simultaneously measured instantaneous brain state. In this case, the neuronal output of the brain affects the input to the brain, thus closing the loop [3].

The combination of electroencephalography (EEG) and transcranial magnetic stimulation (TMS) [4] presents the potential for closed-loop NIBS, which is accentuated by the recent accessibility of cost-effective real-time processors. Although TMS has been around for several decades, it remains the most effective means to non-invasively excite a particular population of cortical neurons, with it being able to do this at a temporal resolution of microseconds and a spatial resolution of millimeters [2,5]. The EEG signal can be theoretically viewed as a lower-dimensional representation of the instantaneous brain state, and the application of TMS could be seen as a vector that results in a new trajectory by altering a spontaneously occurring brain state [6]. Notably, the new state achieved by the TMS pulse is heavily dependent on the precise state at the time of stimulation. This, therefore, leads to the motivation for developing closed-loop brain state-dependent stimulation paradigms.

In the current study, we focus on the most salient feature of the brain state, i.e., the spontaneous oscillatory activity of neuronal networks measured by EEG [7]. Spatially, the state of interest can be determined locally, i.e., the activity of a specific brain area [8], or on a large scale, such as a brain network’s ensemble activity [9]. However, on a temporal scale, brain states can be observed by spectral power changes in a particular frequency band of interest (e.g., event-related desynchronization [ERD]) or perceiving the phase-state of an oscillating cycle. This former method has been effectively employed for both alpha (8–12 Hz) [10] and beta rhythms (16–22 Hz) [11] in brain-machine interfaces that allow stroke patients to perform robot-assisted motor tasks. Methodologically, closing the loop is challenging, not only for the spectral power but also for the instantaneous phase, because the requirement for real-time signal processing necessitates a time resolution of milliseconds; however, such a time resolution has become possible over the past decade [11,12,13]. The implementation of a closed-loop system requires several technically demanding steps: measurement of brain output, signal processing, and tuning/varying of the stimulus. This combination of procedures has become practicable with the latest advances in information technology, which permit intricate calculations to be implemented in real-time using low-cost standard hardware.

Previous benchmark studies [14,15,16] used a genetic algorithm to optimize parameters prior to algorithm deployment. The optimization process needed rigorous computational measures and could not therefore be implemented in real-time. The algorithm proposed in the aforementioned studies may be improved by adopting an online strategy [14] rather than implementing an offline optimization procedure before the actual execution. One alternative is to use the robust method, i.e., wavelet ridge extraction for instantaneous phase estimation [17]. Although this technique is quite useful for highly variable multiple oscillations presented simultaneously, it might be too computationally expensive for real-time implementation. It is possible that real-time implementation may also be restricted by edge effects due to the presence of data in the reverse direction only. Another alternative is the machine learning implemented by McIntosh and Sajda [18], who estimated the EEG phase for real-time applications. Their technique can be used as a substitute for non-causal filtering in offline analysis for phase estimation, but the major drawbacks include the need for preliminary data for training and the risk of unbiased phase estimation. Therefore, an adaptive method is needed to estimate the phase in real-time.

Our previous study [19] presented an adaptive approach for forward prediction of time-series by comparing a conventional Yule–Walker (YW)-based autoregressive model (AR) and a least mean square (LMS)-based AR model. The main aims of the study were to accurately estimate the instantaneous phase of alpha oscillations using the adaptive approach and to compare adaptive and conventional approaches. The present study is an extension of our previous work, i.e., real-time implementations of conventional YW-based AR and adaptive LMS-based AR models. For the current study, we constructed an EEG-triggered visual stimulus closed-loop setup that synchronized the visual stimulus with a specific phase of ongoing alpha oscillations from the occipital cortex. The previous benchmark study [14] used a YW-based AR model to estimate the instantaneous phase of intracranial EEG theta rhythms of only two patients in real-time, whereas a recent study [15] used the same conventional AR model combined with TMS for real-time mu rhythm phase estimation. The current study’s main aim was to check and confirm whether an adaptive LMS-based AR model can be implemented in a real-time closed-loop system, along with a conventional one. Due to technical issues in the DC mode of EEG amplifiers, only data recorded in the AC mode were analyzed for a real-time closed-loop system, which led to a small number of participants. Therefore, this study did not confirm the advantage of the adaptive method over the conventional one.

## 2. Materials and Methods

### 2.1. Implementation of a Closed-Loop System

**Figure 1:**
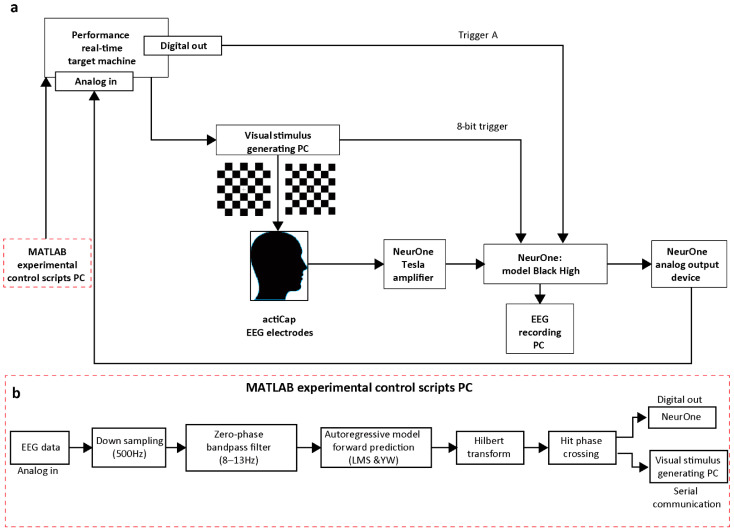
A schematic diagram of the real-time closed-loop system. (**a**) shows an implementation of a closed-loop brain state-dependent visual stimulation setup comprising electroencephalography (EEG), real-time signal processing, and triggered visual stimulation. The visual stimulation is locked to the instantaneous phase of the recorded EEG signal in the alpha band either at the peak or the trough. (**b**) shows sequential steps for time-series forward prediction implemented through MATLAB experimental control scripts PC via four distinct Simulink real-time models (Yule–Walker (YW) peak, YW trough, least mean square (LMS) peak, LMS trough). Raw EEG data were downsampled first, followed by finite impulse response (FIR) bandpass filtering. Coefficients of the autoregressive (AR) models were calculated, and the EEG signal was forward predicted. After time-series forward prediction based on YW/LMS methods, the instantaneous phase (at time-zero”) was estimated using Hilbert transform. The visual stimulation was then triggered when a pre-set phase (peak or trough) condition was met.

We propose an experimental setup that prolongs current approaches by closing the loop between EEG signals (representing the instantaneous brain state) and visual stimulation. The timing of visual stimulation is locked to the online-detected instantaneous phase of the EEG alpha-band signal (peak and trough phases). An implementation of a real-time closed-loop system is shown in Figure 1. EEG signals are acquired using an actiCAP slim (BrainProducts GmbH, Gilching, Germany) electrode system. Two 24-bit 32-channel Tesla EEG amplifiers (NeurOne; Bittium Biosignals Ltd., Kuopio, Finland) are used for the EEG recordings, with data recorded in the AC mode at a sample rate of 20 kHz for subsequent analysis. The amplifiers’ analog output device is configured to recreate a filtered and an amplified analog signal from a user-selectable set of 16 amplifier channels covering the occipital cortex. Of the channel subset, O_z_ is analyzed by a real-time system. A MATLAB experimental control scripts PC is connected to the Performance real-time target machine (Speedgoat GmbH, Liebefeld, Switzerland), which receives input signals from a 24-bit analog input module (IO109) and sends TTL signal output to a digital output module (IO203 with 64 TTL channels). The digital output module further sends the TTL trigger signal from the Performance real-time target machine to the NeurOne model Black High (Bittium Biosignals Ltd., Kuopio, Finland), with it being referred to here as Trigger A. An additional 8-bit trigger is simultaneously sent from the Performance real-time target machine to the visual stimulus generating PC via serial port (RS232), which further sends it to the NeurOne model Black High for subsequent data analysis. A real-time data acquisition system (Figure 1b) utilizing a Performance real-time target machine runs in parallel on a dedicated target PC (SN4200, IO10; Speedgoat), digitally processing and archiving the raw EEG data through the implementation of a Simulink real-time model (MathWorks Inc., Natick, MA, USA, 2018a) for each scenario (YW peak and trough, LMS peak and trough). The time lag due to the Performance real-time target machine is approximately 10 ms, while the time lag due to the NeurOne is approximately 4 ms.

### 2.2. Algorithm

The ultimate goals of this study were the real-time phase estimation of alpha rhythms and phase-dependent triggering. The instantaneous phase prediction algorithm can be divided into four distinct parts: YW peak prediction, YW trough prediction, LMS peak prediction, and LMS trough prediction. Each part was further divided into the following sequential steps, except for step 4, which differs between the YW-based and LMS-based AR models. A distinct Simulink real-time model (“MATLAB experimental control scripts PC” block in Figure 1, part b) was designed for each one of the four methods and implemented in the Performance real-time target machine.

1. In each Simulink model, the raw EEG data are received as analog input via IO109 at a sample rate of 2 kHz and are downsampled to 500 Hz.
2. The data are then delayed by 500 samples, and the mean of the data is calculated and subtracted from the original data. The data are then sent to the next step for filtering.
3. The third step implements bandpass filtering. A two-pass finite impulse response (FIR) bandpass filter (filter order 128) with an 8–13 Hz frequency range is applied to the data, and the edges are removed.
4. The fourth step is forward prediction. After trimming 85 samples from both sides, the remaining 330 samples are then used for forward prediction (85 samples). The YW forward prediction algorithm predicts the future and computes coefficients using Yule–Walker equations, whereas the LMS forward prediction algorithm uses an adaptive method to compute coefficients and then uses them in the AR equation. This step results in a predicted signal as an output. The model order for both methods is 30.
5. The Hilbert transform is performed on resulting forward-predicted EEG data to determine the instantaneous phase at “time-zero”.
6. The zero-phase crossing (a predetermined phase is crossed, with 0 and pi rad portraying positive and negative peaks, respectively) is monitored online, and a TTL signal is sent from the Performance real-time target machine via digital output module (IO203) and serial port (RS232). The Performance real-time target machine sends the TTL signal to the EEG recording PC via IO203, while at the same time, the TTL signal is sent via RS232 to the visual stimulus generating PC.

The next subsections briefly explain the AR model, LMS, instantaneous phase, and phase-locking factor.

### 2.3. Autoregressive (AR) Model

AR modeling has been effectively utilized in various EEG analysis applications, such as forecasting [20,21], segmentation, and speech analysis [22]. AR modeling shows great results for the power spectrum estimation of short-duration EEG data because of its low vulnerability toward false results [20]. Numerous algorithms can be used to calculate the AR model coefficients, including the Yule–Walker and Burg lattice algorithms.

An AR model of order *K* is a random process defined as follows [14]:

(1) $$x\left(t+1\right)=\overset{K-1}{\underset{k=0}{\sum }}\alpha_{k\;}x\left(t-k\right)+\varepsilon_{t},$$

where $\alpha_{0}$, …, $\alpha_{K-1}$ are the coefficients of the AR model; K is the model order; and $\varepsilon_{t}$ is white noise.

### 2.4. Least Mean Square (LMS)

In 1960, Hoff and Widrow developed the LMS algorithm. LMS uses a stochastic gradient method to solve the least square issue. The equations that establish the adaptive LMS algorithm are defined as follows [23]:

(2) $$X\left(t\right)={\left[x\left(t\right),\;x\left(t-1\right),\;x\left(t-2\right),\ldots ,x\left(t-K+1\right)\right]}^{T},$$

(3) $$A\left(t\right)={\left[a_{0}\left(t\right),\;a_{1}\left(t\right),\ldots ,a_{K-1}\;\left(t\right)\right]}^{T},$$

(4) $$y\left(t\right)=A^{T}\left(t\right)X\left(t\right),$$

(5) e(t)=x(t+1)−y(t),

(6) A(t+1)=A(t)+2μe(t)X(t),

where x(t) is an input signal at sample t, y(t) is output, e(t) is an error, A(t) is the filter weight, μ is the step size, and K is the filter order. The bold variables represent vectors.

For simplicity, in the remaining text, we will use YW to refer to the YW-based AR model and LMS to refer to the LMS-based AR model.

### 2.5. Instantaneous Frequency, Phase

To calculate the instantaneous phase, the analytic signal is created by joining the original data with its Hilbert transform [24]. The analytic signal $z_{x}\left(t\right)$, which is a complex signal of time *t* can be created as follows:

(7) $$z_{x}\left(t\right)=x\left(t\right)+jH\left\{x\left(t\right)\right\},$$

where x(t) is the real signal, and H{x(t)} is the Hilbert transform of the real signal, which is defined as follows:

(8) $$H\left\{x\left(t\right)\right\}=\frac{1}{\pi \;}\;P.V.\int_{-\infty }^{\infty }\frac{x\left(\tau \right)}{t-\tau }\;d\tau ,$$

where P.V. indicates Cauchy’s principal value. The instantaneous phase of the signal can be found from the complex analytic signal as follows:

(9) $$\theta \left(t\right)=\arg z_{x}\left(t\right).$$

### 2.6. Participants

A total of nine volunteers (three males and six females; mean age 32.1 years ± 6.6 (standard deviation [SD])) with normal or corrected-to-normal vision were recruited to this study and provided informed consent for the EEG experiments. The study was approved by the ethics committee of RIKEN. Data from the first three participants were recorded using the DC mode of the Tesla amplifier, while data from the rest of the participants were recorded using the AC mode. The participant data recorded using the DC mode was noisier than that recorded using the AC mode, which resulted in quite low amplitude output signals. The AC mode has a higher signal-to-noise ratio than the DC mode and uses a low pass filter of 0.16 Hz, which leads to higher amplification of data for subsequent analysis. The prediction accuracy was highly affected by the signal-to-noise ratio, and therefore only the participant data collected using the AC mode were used for further analyses. A spectral analysis was performed on the remaining six participants to estimate the power in the alpha rhythm range (8–13 Hz).

### 2.7. Experiment

**Figure 2:**
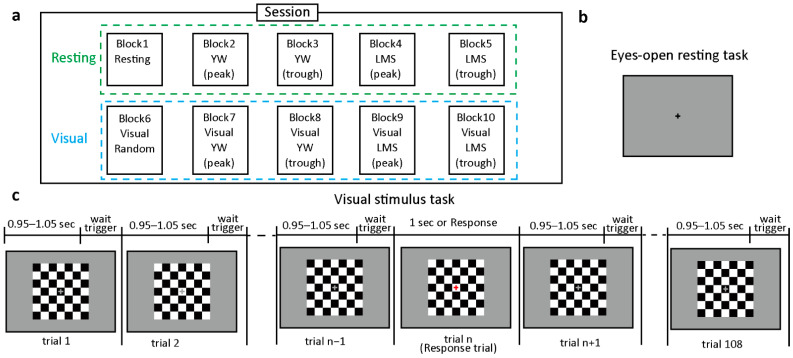
Overview of the experimental sessions and trials. (**a**) shows a session divided into resting (green) and visual (blue) tasks, each with five blocks. The instantaneous phase prediction algorithm was implemented utilizing four distinct models: YW peak, YW trough, LMS peak, and LMS trough. “Peak” means a positive peak or 0 rad, while “trough” depicts a negative peak or pi rad. (**b**) shows an eye-open resting condition. (**c**) shows trials of the visual stimulus condition. The visual stimulus task includes passive and response trials. The stimulus comprised of a checkerboard with a gray fixation cross at the center in passive trials. While response trials are shown with a red fixation cross at the center. Each trial took an average of 1.05 s.

The experiment incorporated visual stimulation blocks and eyes-open resting blocks. Participants were asked to avoid eye blinks, eye movements, and jaw clenching. The whole experiment was divided into two sessions, each with ten blocks presented in random order, with these ten blocks consisting of five resting and five visual blocks. In both sessions, the ten blocks were linked with conditions, namely Resting, Visual Random, Resting YW (peak, trough), Resting LMS (peak, trough), Visual YW (peak, trough), and Visual LMS (peak, trough), as shown in Figure 2a. Results were analyzed from blocks 2–5 and blocks 7–10. The total experiment took 1 h and 10 min, including small breaks between blocks. There were 90 trials each block for the resting condition and 108 trials for the visual stimulation condition, as shown in part c of Figure 2. The visual experiment consisted of normal trials and response trials. Visual stimuli were shown on an LCD monitor (BenQ XL2420; BenQ Corporation, Taipei, Taiwan; refresh rate: 144 Hz; resolution: 1920 × 1080), with a chin rest placed 100 cm from the monitor being used to maintain head position. The checkerboard stimuli (visual angle of 8.8°) consisted of 49 black-and-white squares (7 by 7) with a fixation cross at the center. The color of the grids was temporally modulated between black and white (luminance of black: 9.18 cd/m^2^; white: 152.2 cd/m^2^). The fixation cross was colored gray in regular trials and red in response trials. Participants were instructed to press the left mouse button as soon as they saw a red fixation cross. The visual and resting tasks were implemented using NBS Presentation Version 20.0 (Neurobehavioral Systems Inc., Albany, CA, USA). In addition to the visual experiment, EEG signals were also measured for the resting scenario, while participants rested with their eyes open looking passively at the fixation cross displayed in the center of the screen part b Figure 2. 

### 2.8. EEG Recording and Preprocessing

The 63-channel EEG signals were recorded at a sampling rate of 20 kHz using two Tesla amplifiers and an actiCAP slim EEG cap. Online low and high cutoff frequencies for the EEG amplifier were set to 0.16 Hz and 3500 Hz, respectively. Electrodes were positioned according to the 10/10 system, with electrode AF_z_ as the ground electrode and the left earlobe as the reference electrode. For offline analysis, EEG signals were re-referenced to the average of the right and left earlobe and downsampled to 500 Hz. Only the downsampled signal was used to calculate the phase-triggered response (PTR), whereas, for the phase-locking factor (PLF) and instantaneous phase calculation, a two-pass FIR bandpass filter (8–13 Hz) with a filter order of 128 was applied to the EEG signals. All analysis was performed in MATLAB R2018a (MathWorks Inc., Natick, MA, USA) using EEGLAB [25] and a personalized script.

### 2.9. Statistical Analysis

All statistical analyses were performed using MATLAB and the Statistics and Machine Learning Toolbox, with *p* < 0.05 being set as the level of statistical significance.

## 3. Results

The main aim of the current study was to implement an adaptive LMS model in real-time as well as a conventional YW model for individual participants. We divided the results into two subsections: “resting” (focusing on resting with eyes open) and “visual” (based on the visual stimulus task). To check the performance of both YW and LMS methods, the PLF at time-zero was assessed.

### 3.1. Phase-Locking Factor

The PLF was defined as follows:

(10) $$PLF=\frac{1}{N}\left|\;\overset{N}{\underset{n=1}{\sum }}e^{j\theta_{n}}\right|,$$

where $\theta_{n}$ is the instantaneous phase at time-zero for the *n*th trial, and *N* is the total number of trials. A *PLF* closer to zero indicates high phase variability across trials, while a *PLF* closer to 1 depicts all trials as having the same phase. It should be noted that the phase variance is 1-*PLF* [26].

The statistical significance of the *PLF* can be tested by a Rayleigh test to calculate *ZPLF* [26,27], which is Rayleigh’s *Z* value computed using *PLF* as follows:

(11) $$ZPLF=N{\left(PLF\right)}^{2}.$$

To assess the statistical significance of the participant-averaged *ZPLF* values, the value was corrected to $ZPLF_{all}$:

(12) $$ZPLF_{all}=\frac{1}{\sqrt{M}}\overset{M}{\underset{m=1}{\sum }}ZPLF_{m},$$

where *M* is the number of participants [28].

To evaluate the difference between YW and LMS within each participant, we also examined Watson’s U^2^ test for each of the two conditions (resting and visual), according to the method proposed by Persson [29]. If the calculated U^2^ is larger than the critical value, the two sample circular distributions differ significantly from each other. For the current study, the critical value U^2^ (∞, ∞; *p* < 0.05) = 0.187. As this test compares both the phase variance and the average of the phase angular data, the effects of the difference in average phase angles were removed by shifting the phase according to the following [30]:

(13) $$\theta_{new}=\theta -\varphi ,$$

(14) $$C=\frac{1}{N}\overset{N}{\underset{n=1}{\sum }}\cos\theta_{n},$$

(15) $$S=\frac{1}{N}\overset{N}{\underset{n=1}{\sum }}\sin\theta_{n},$$

where θ is the vector of instantaneous phases at zero ms, and $\left[\theta_{1},\;\cdots ,\;\theta_{N}\right]$, $\varphi =tan^{-1}\left(S/C\right)$, and $\theta_{new}$ are used in the calculation of Watson’s U^2^ test.

Using this transformation, we can compare the differences in phase variance between the two circular distributions.

### 3.2. Phase-Triggered Response (PTR)

PTR is defined as the grand-average of triggered EEG signals from distinct trials within each participant.

(16) $$PTR\left(s\right)=\frac{1}{N}\overset{N}{\underset{n=1}{\sum }}S_{n}\left(s\right),$$

where $S_{n}$ is the downsampled EEG signal for the *n*th trial as a function of the sample point *s* within each trial extracted based on the trigger at time zero, which are generated by the phase prediction methods. *s* ranges between 0 and 1000 centered around time-zero, N is the total number of trials for each participant.

PTR is calculated similarly to event-related potentials (ERP), but it does not depend on the external stimulus (such as visual or auditory stimuli) and uses a generated trigger based on the EEG phase. It is a measure for checking the prediction performance.

### 3.3. Resting Conditions

**Figure 3:**
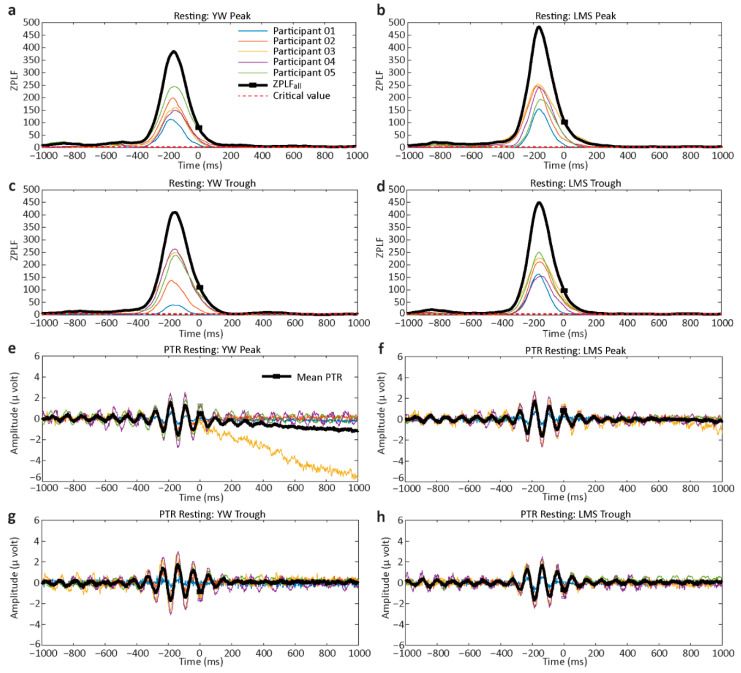
Rayleigh’s Z value (ZPLF) and phase-triggered response (PTR) for the resting task for each participant. The bold lines depict ZPLF_all_ or mean PTR. (**a**) ZPLF results for the individual participants for the YW method peak condition. (**b**) ZPLF results for the LMS method peak condition. (**c**) ZPLF results for the YW trough condition. (**d**) ZPLF for the LMS trough condition. (**e**) PTR for the YW peak condition. (**f**) PTR for the LMS peak condition. (**g**,**h**) PTR for the YW and LMS trough conditions, respectively. The red dashed lines in (**a**–**d**) shows the critical value of 2.995. ZPLF larger than this critical value is considered to be statistically significant. The black lines in e–h indicate the mean PTR averaged across five participants. The small black square depicts the time-zero.

The results of ZPLF and PTR for the resting condition are shown in Figure 3. ZPLF and PTR are shown individually for the five participants. The bold black lines in Figure 3a–d indicate ZPLF_all_. For a number of trials >60, a ZPLF > 2.995 (which is called the critical value) is considered statistically significant. ZPLF_all_ is also statistically significant if it exceeds the critical value. The small square box on the ZPLF_all_ line represents time-zero. In parts a–d, ZPLF_all_ crosses the critical value indicated as a dotted red line. We found that ZPLF and ZPLF_all_ were statistically significant for all participants except for the YW trough condition in participant P01. Our results are in accord with previous studies showing a ZPLF decrease when time increases [19,31]. Figure 3e–h shows the PTR for individual participants. In the PTR plots, the squares for time-zero are observed at the peak for the peak condition and the trough for the trough condition. The bold black lines show the mean PTR.

**Figure 4:**
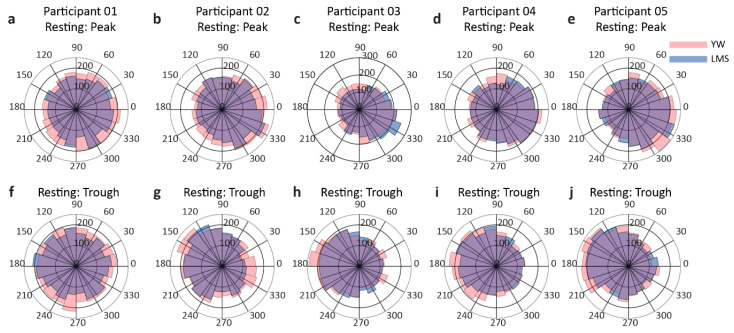
Rose plots for the resting conditions for each participant. The upper row (**a**–**e**) shows the rose plots for each participant’s peak condition, while the lower row (**f**–**j**) depicts the rose plot for the trough condition for each participant. The peach color indicates the YW method, while purple indicates the LMS method. The violet color depicts the overlapping region. For the peak condition, the rose plots are inclined towards 0 rad and for the trough condition, the rose plots show an inclination towards pi rad.

Rose plots for each participant are shown in Figure 4. For the peak condition, these rose plots show an accumulation of values toward 0 rad, while for the trough condition, the rose plots show an accumulation toward pi rad. The summarized results of PLF and ZPLF and their mean ± SD are shown in Table 1. The bold values indicate significantly higher ZPLF compared to a critical value of 2.995. Moreover, the ZPLFs of all participants crossed the critical value at time-zero, except for the YW trough condition of participant P01, as shown in Figure 3c. The mean angle in radians and Watson’s U^2^ test results are shown in Table 2. The bold values indicate where the calculated U^2^ values are greater than the critical value, and the differences in the two-phase variances are statistically significant. For participants P01 and P02, the LMS trough performed better than the YW trough. For participant P03, the LMS peak performed better than the YW peak condition. For participant P04, the YW trough surpassed the LMS trough. No significant difference was shown in the results of participant P05 indicating both methods YW and LMS performed equally at time-zero.

Taken together, the results suggest that we succeeded in outputting triggers targeting specific phases of alpha oscillations in a real-time implementation under resting conditions, doing this with both YW-based and LMS-based AR models.

### 3.4. Visual Condition

**Figure 5:**
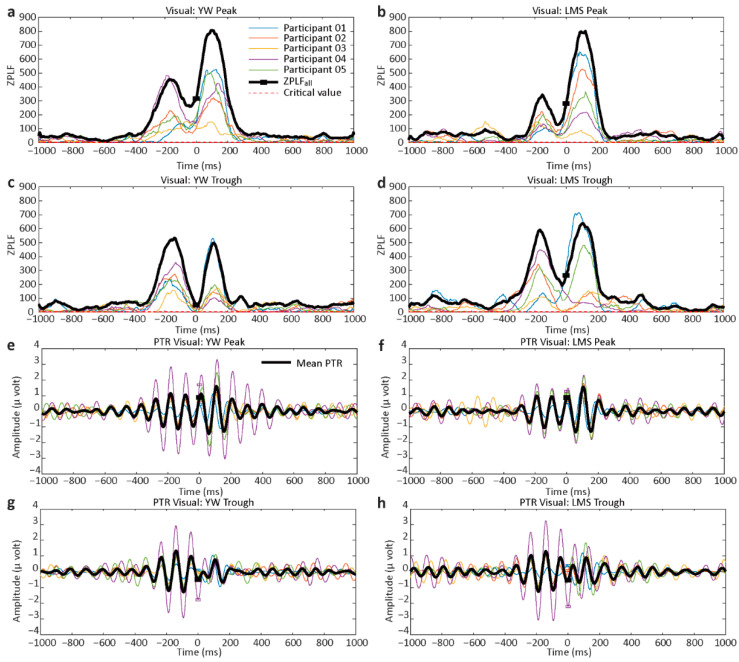
ZPLF and PTR for the visual task. (**a**–**d**) show ZPLF results for both YW and LMS methods with peak and trough conditions, while (**e**–**h**) show the phase-triggered response (PTR) for YW and LMS methods for the peak and trough conditions. ZPLF shows a second peak around 100 ms for the visual task. The small black square shows time-zero. The bold black signals in (**e**–**h**) show the mean PTR averaged across five participants. The red dashed lines in (**a**–**d**) shows the critical value of 2.995. ZPLF larger than this critical value is considered to be statistically significant.

**Figure 6:**
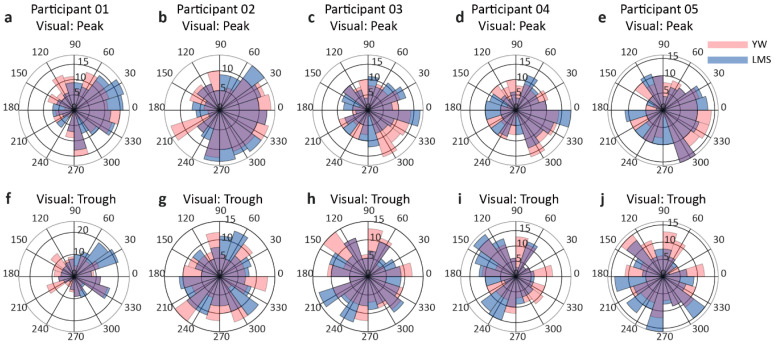
Rose plots for the visual condition. The peak conditions for each participant are shown in the first row (**a**–**e**), while the trough conditions are depicted in the second row (**f**–**j**). For the peak condition, the rose plots are inclined towards 0 rad and for the trough condition, the rose plots show an inclination towards pi rad. (**f**) shows no tendency towards any phase angle.

The results of ZPLF and PTR for the visual condition are shown in Figure 5. We observed two peaks in ZPLF for the visual condition, with the second peak corresponding to the visual response around 100 ms. The small black square in Figure 5 shows time-zero. Rose plots for the visual condition are presented in Figure 6. For the peak condition, the rose plots are somewhat inclined toward the right side (0 degrees), but for the trough condition, there is not a clear inclination toward the left side. For participant P01, the rose plots do not show any leaning toward the left side. The summarized PLF, ZPLF, mean angle, and Watson’s U2 test results for the visual task are shown in Table 3 and Table 4. All participants showed significant ZPLF values for each method and each condition. No significant difference was observed for any condition in participants P04 and P05. For participants P01 and P03, the LMS trough surpassed the YW trough, but for participant P02, the YW trough was better than the LMS trough, as shown by the bold Watson U2 test values.

The results suggest that we succeeded in giving visual stimulation targeting specific phases of alpha oscillations in a real-time implementation, doing this with both YW-based and LMS-based AR models and that we observed the stimulation-induced brain responses.

The results of both resting and visual tasks in terms of percentage and the total number of participants who showed the significant ZPLF are summarized in Table 5. ZPLF value > 2.995 is considered to be significant. Significant ZPLF means that we achieved the desired result of outputting the triggers targeting a specific phase of alpha oscillations. For the resting peak condition, all participants showed significant ZPLF values for both methods. For the trough condition, all participants indicated the significant ZPLF for LMS and only one participant did not show the significant ZPLF value for the YW method. In the visual task, results indicate that all participants in both methods and all conditions crossed significant ZPLF value. Taken together, all the results indicate that we succeeded in outputting the triggers targeting specific phases of alpha oscillations in a real-time implementation, doing this with both YW-based and LMS-based AR models except for one participant in one condition.

## 4. Discussion

Our study implemented, for the first time, real-time EEG phase-dependent triggers for visual stimulation using EEG signals from the human occipital cortex (channel Oz). These triggers were based on a novel adaptive LMS-based AR model as well as the conventional YW-based AR model. The primary purpose of our study was to investigate the possibility of a real-time implementation of a closed-loop system based on the adaptive LMS-based method, which we previously proposed and demonstrated by analyzing offline data [19]. Earlier studies employed the complex wavelet transform [32] or the Hilbert–Huang transform [33,34] to extract the frequency and phase information from EEG. However, the application of these methods is limited because of the future prediction of nonstationary data. Although the conventional YW-based AR model can resolve the EEG time-series’ prediction, it presumes the stationarity of signals over a certain period and is, therefore, less appropriate for closed-loop and real-time applications for nonstationary time-series data such as EEG. By contrast, our previously proposed adaptive method depends on recurrent updates to cater for the non-stationarity of EEG signals, thereby making adjustments to dynamic changes while predicting the future signal. The results of this proof-of-concept study provide empirical evidence that the adaptive method is implementable in a real-time situation.

We found individual differences in the performance of the closed-loop system and results are presented individually at time-zero. In the resting condition, all participants showed significant ZPLF for both methods (peak and trough conditions), except for the trough condition in the YW-based method for participant P01. In the visual conditions, all participants showed significant ZPLF for each condition in both methods. Participant P05 showed no significant difference between any of the conditions, indicating that both methods performed equally for this participant. These results suggest that although there are individual differences in the predictability of EEG phases, the proposed method performed well in outputting the stimulation triggers for most participants.

In a previous study [19], we showed the advantage of our LMS-based method over the YW-based method in alpha-band EEG phase prediction using offline analyses. However, we could not provide evidence of the superiority of the adaptive LMS-based method at time-zero because of the small number of participants, which led to difficulty in making a clear comparison. Although the real-time implementation of the adaptive method was our aim, one limitation of this study is that technical issues in the DC mode of the EEG amplifiers meant that only data from five participants for whom the AC mode was used were analyzed for the real-time closed-loop system. Therefore, further studies will need to clarify whether the adaptive proposed method outperforms the conventional non-adaptive method in a real-time implementation. This is important because there can be technical issues that are specific to real-time implementation.

Our results show that we succeeded in measuring brain responses with respect to triggered visual stimulation. Although our study applied visual stimulation only, there is the possibility of using the adaptive method to trigger TMS and other NIBS techniques.

One advantage of our proposed method is the low computational cost. Some prior studies focused on using machine learning methods for phase estimation, and a variety of machine learning techniques, particularly deep learning, have been applied in brain-computer interface (BCI) systems [18,35,36]. The main drawback of such procedures is the demand for preliminary data for training prior to the principal experiment. Because of the absence of future information in real-time phase estimation, the trained filters rely on the properties and quality of the signal, and as a consequence, the technique does not perform unbiased phase estimation. Deep learning techniques, while being highly efficient, still need an abundant amount of data for training and a large amount of processing power, and are thus expensive to implement. By contrast, our proposed method does not require extensive training and computational cost.

We propose that our method can be applied not only to targeting in basic neuroscience (for example, the functional role of neural oscillations) but also to clinical fields. In the last decade, substantial progress has been made in invasive brain stimulation that dynamically responds to the presence of divergent neural activity [37,38]. For example, in a small group of Parkinson’s disease patients, deep brain stimulation resulted in clinical improvements of approximately 30% compared to a standard open-loop system [39]. When required, a closed-loop stimulation device might deliver stimulation more proficiently by performing stimulation only when brain function is damaged or shows abnormal neural activity [40] and synchronizing each stimulus with the patient’s instantaneous brain state. State-dependent brain stimulation has therapeutic potential for brain disorders like epilepsy, schizophrenia, Parkinson’s disease, and stroke. Further studies will involve implementing the adaptive method in a TMS-EEG real-time scenario and exploring new prospects for alpha and other oscillations.

## 5. Conclusions

We have presented here a real-time closed-loop system that implements both an adaptive LMS-based AR model and a conventional YW-based AR model. This system consists of a time-series forward prediction and phase-locked visual stimulation. Brain state-dependent, EEG triggered visual stimulation was applied and synchronized with the EEG peaks and troughs of alpha oscillations in both a resting state (open eyes) and a visual task. Our results indicate that we succeeded in outputting triggers targeting specific phases of alpha oscillations in a real-time implementation, doing this with both YW-based and LMS-based AR models. Our adaptive approach for EEG phase estimation relies on predictions of instantaneous alpha oscillations, does not depend on the knowledge of the exact stochastic signal, and tracks the variation in the EEG signal by dynamically adjusting its coefficients. Although we have focused only on alpha oscillations, this system can also be employed to analyze oscillations in other frequency bands. This novel implementation may lead to EEG instantaneous phase prediction with low computational cost and provide versatile applications in basic and clinical neurosciences.

## Figures and Tables

**Table 1:** Summary of the results of the resting condition. The number of trials, PLF, and ZPLF at time-zero are shown.

Resting												
ID	Number of Trials				PLF				ZPLF			
	YW Peak	LMS Peak	YW Trough	LMS Trough	YW Peak	LMS Peak	YW Trough	LMS Trough	YW Peak	LMS Peak	YW Trough	LMS Trough
P01	3598	3156	3710	3276	0.057	0.059	0.019	0.047	**11.872**	**11.102**	1.432	**7.378**
P02	3491	3093	3433	3075	0.104	0.103	0.075	0.126	**37.831**	**32.895**	**19.778**	**51.678**
P03	3192	3038	3099	2993	0.090	0.171	0.155	0.149	**26.283**	**89.706**	**74.474**	**66.55**
P04	3230	3089	3268	3053	0.101	0.125	0.146	0.107	**33.569**	**48.528**	**70.241**	**35.50**
P05	3326	3159	3340	3139	0.146	0.122	0.155	0.133	**71.307**	**47.381**	**80.566**	**55.752**
Mean	3367.4	3107	3370	3107.2	0.100	0.116	0.110	0.113	36.172	45.922	49.298	43.371
SD	173.068	50.955	226.005	107.843	0.031	0.040	0.060	0.039	21.978	28.758	36.099	23.005

**Table 2:** Mean angle and Watson U^2^ test results at time-zero for the resting condition.

Resting						
ID	Mean Angle (rad)				Watson U^2^	
	YW Peak	LMS Peak	YW Trough	LMS Trough	YW vs. LMS Peak	YW vs. LMS Trough
P01	−0.475	−0.154	−3.009	2.580	0.059	**1.125**
P02	−0.228	−0.108	2.821	2.761	0.054	**0.273**
P03	−0.350	−0.369	2.923	2.956	**0.570**	0.099
P04	−0.271	−0.337	2.920	2.613	0.078	**0.207**
P05	−0.216	−0.333	2.872	2.827	0.887	0.064
Mean	−0.297	−0.260	2.961	2.747		

**Table 3:** Summary of the results of the visual condition. The number of trials, PLF, and ZPLF at time-zero are shown.

Visual												
ID	Number of Trials				PLF				ZPLF			
	YW Peak	LMS Peak	YW Trough	LMS Trough	YW Peak	LMS Peak	YW Trough	LMS Trough	YW Peak	LMS Peak	YW Trough	LMS Trough
P01	3780	3671	3788	3646	0.204	0.264	0.062	0.302	**158.59**	**255.83**	**14.760**	**333.357**
P02	3772	3553	3776	3575	0.166	0.207	0.078	0.047	**105.10**	**153.43**	**23.016**	**8.146**
P03	3630	3420	3760	3385	0.176	0.107	0.037	0.071	**112.73**	**39.500**	**5.326**	**17.461**
P04	3745	3762	3472	3461	0.194	0.150	0.141	0.193	**142.02**	**85.397**	**69.912**	**128.927**
P05	3774	3549	3776	3588	0.224	0.162	0.035	0.170	**190.26**	**93.699**	**4.871**	**103.984**
Mean	3740.2	3591	3714.4	3531	0.193	0.178	0.071	0.157	141.74	125.57	23.577	118.384
SD	63.057	130.47	135.870	105.624	0.022	0.059	0.043	0.102	34.720	83.342	26.962	131.216

**Table 4:** Visual condition results. The mean angle and Watson U^2^ test results for the visual condition at time-zero are shown.

Visual						
ID	Mean Angle (rad)				Watson U^2^	
	YW Peak	LMS Peak	YW Trough	LMS Trough	YW vs. LMS Peak	YW vs. LMS Trough
P01	0.478	0.158	0.708	0.449	0.054	**0.338**
P02	−0.481	−0.407	−2.030	−1.090	0.031	**0.951**
P03	−0.169	−0.117	2.643	−2.112	0.046	**0.554**
P04	−0.655	−0.603	−2.810	−3.067	0.064	0.069
P05	−0.883	−0.657	−2.673	−2.167	0.093	0.151
Mean	−0.355	−0.327	−2.918	−1.882		

**Table 5:** Overview of resting and visual task results. Percentage and the total number of participants showing a significant ZPLF value for each condition.

Total Participants = 5	Resting				Visual			
	YW Peak	LMS Peak	YW Trough	LMS Trough	YW Peak	LMS Peak	YW Trough	LMS Trough
Participants	5/5	5/5	4/5	5/5	5/5	5/5	5/5	5/5
Percentage	100%	100%	80%	100%	100%	100%	100%	100%

## Data Availability

The data and code used to support the findings of this study are available from the corresponding author upon reasonable request.
